# PIK3R1 as the Hidden Hand in Arrhythmogenic Right Ventricular Cardiomyopathy Inflammation: Weaving Transcriptomic Signatures with Structural Therapeutic Insights

**DOI:** 10.3390/ph18121873

**Published:** 2025-12-09

**Authors:** Nazia Azim, Ashwag Saleh Alsharidah, Mansour Alsharidah, Nadeem Khan, Sajjad Ahmad

**Affiliations:** 1 Department of Computer Science, Abdul Wali Khan University, Mardan 23200, Pakistan; 2 Department of Physiology, College of Medicine, Qassim University, Buraydah 52571, Saudi Arabia; 3 Department of Health and Biological Sciences, Abasyn University, Peshawar 25000, Pakistan

**Keywords:** arrhythmogenic right ventricular cardiomyopathy (ARVC), transcriptomics analyses, bioinformatics, drug development, drug efficacy, molecular docking simulation

## Abstract

**Background**: Arrhythmogenic right ventricular cardiomyopathy (ARVC) is a congenital cardiac disorder, but its severity has been increasingly linked to inflammatory processes. This study aimed to investigate gene expression profiles in ARVC to identify genes potentially driving inflammation in affected individuals. **Methods**: Publicly available gene expression datasets comprising 12 ventricular tissue samples from six clinically confirmed ARVC patients (paired left and right ventricular biopsies) and 12 ventricular samples from six non-failing donor hearts were analyzed to identify differentially expressed genes. Immune infiltration was assessed to determine the proportions of immune cells in the ARVC condition. Correlation analysis between immune cell proportions and gene expression profiles was further performed to identify genes linked with inflammation-specific immune cells. Functional enrichment analysis of associated genes was performed to pinpoint the key involvement of genes in different inflammatory-specific pathways. Finally, the key gene associated with inflammation-specific immune cells and its active involvement in inflammatory pathways was further subjected to molecular docking against a curated library of marine-derived phytochemicals, followed by 100 ns molecular dynamics simulations to evaluate ligand stability. **Results**: A total of 141 significantly upregulated genes were identified in ARVC. Immune infiltration analysis revealed elevated proportions of regulatory T cells, CD8^+^ T cells, plasma cells, M2 macrophages, resting mast cells, and activated NK cells in the ARVC phenotype, indicating an immunologically active microenvironment. Correlation analysis identified four genes—LIFR, SCN2B, RGCC, and PIK3R1—showing significant positive associations with these immune cells. Functional enrichment analysis highlighted PIK3R1 (LogFC > 2.00) as a central regulator in the PI3K/AKT and mTOR pathways, which govern immune activation, cell survival, and fibrosis. Molecular docking identified two marine compounds, CMNPD18967 and CMNPD756, with strong binding affinities (−5.9 and −5.7 kcal/mol, respectively). Molecular dynamics simulations confirmed stable ligand binding within the PIK3R1 active site. **Conclusions**: PIK3R1 emerges as a key inflammation-associated gene in ARVC, with strong involvement in immune-regulatory pathways. Marine-derived phytochemicals CMNPD18967 and CMNPD756 demonstrate promising inhibitory potential through stable interaction with PIK3R1. While these findings present potential anti-inflammatory leads, validation in larger clinical cohorts and experimental models is essential to confirm translational applicability.

## 1. Introduction

Arrhythmogenic right ventricular cardiomyopathy (ARVC) is a rare hereditary heart disorder characterized by the progressive replacement of right ventricular (RV) myocardium with fibrofatty tissue, affecting approximately 1 in 5000 people in the general population, and accounts for 10% of the sudden deaths in young individuals [1,2,3]. This condition is caused by genetic mutations in the genes encoding the desmosomal proteins, crucial for cell–cell adhesion in cardiac muscles. These mutations lead to compromised structural integrity of cardiac myocytes that predispose the heart to electrical instability, ventricular arrhythmias, syncope, and fibrosis [4,5].

ARVC can be difficult to diagnose due to its diverse clinical presentation, extremely variable intra- and inter-family expressivity, numerous host protein mutations, and inadequate penetrance. Yet there is no gold-standard test to diagnose ARVC; instead, it is determined by a scoring system with “major” and “minor” criteria based on the presence of a number of defects in right ventricular (RV) morphology and function, characteristic tissue pathology, characteristic depolarization/repolarization ECG abnormalities, typical arrhythmias, family history, and genetic test results [6,7].

Inflammation is considered a major key factor in mitigating the severity of ARVC phenotype and other cardiovascular disorders [8,9]. This concept is supported by the numerous histopathological findings, where patchy inflammatory infiltration was found in up to two-thirds of affected heart tissues [10]. ARVC patients exhibit elevated levels of circulating pro-inflammatory cytokines, including TNF-α, IL-6, and IL-1β. These elevated cytokine levels correlate with disease severity and arrhythmic burden, suggesting a link between systemic inflammation and ARVC progression [11]. Furthermore, in the most severe forms of the illness with biventricular involvement, a postmortem examination of 36 hearts with ARVC revealed inflammatory T lymphocyte infiltrates, indicating inflammation as a critical factor in determining the disease’s severity [12].

Given the central role of inflammation in modulating ARVC severity, targeting pro-inflammatory signaling pathways represents a promising therapeutic avenue. Currently, treatment strategies for ARVC focus primarily on symptom management, including anti-arrhythmic drugs, implantable cardioverter-defibrillators (ICDs), and lifestyle modifications to reduce physical exertion. However, recent preclinical studies suggest that immunosuppressive agents and anti-inflammatory compounds may attenuate disease progression by reducing myocardial inflammation and fibrosis [13]. Despite these advances, no inflammation-targeted therapies have yet been approved for ARVC, highlighting the need for further molecular investigations and drug discovery efforts.

In this context, the present study aims to identify key inflammation-associated genes differentially expressed (DE) in ARVC patients, using integrative bioinformatics approaches. Furthermore, we employ a therapeutic drug discovery pipeline comprising molecular docking and simulation analyses to identify potential small molecules capable of inhibiting inflammation-induced targets. This combined diagnostic and therapeutic strategy not only enhances our understanding of ARVC pathophysiology but also offers a potential route for targeted intervention aimed at mitigating inflammation-driven cardiac damage.

## 2. Results

### 2.1. Gene Expression Profiles Differentiate ARVC Patients from Healthy Individuals

**Figure 1:**
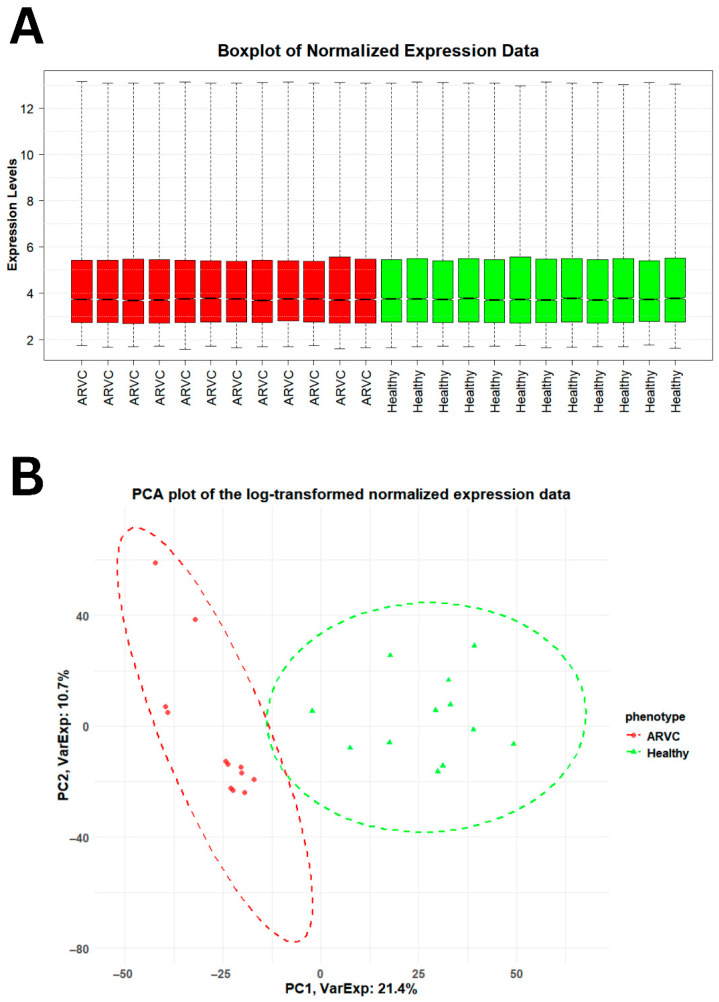
Accessing the distribution and variability of gene expression data across healthy and ARVC. (**A**) The boxplot showed the distribution of gene expression data across the phenotypes. The distribution of 50% of the gene expression across both the phenotypes lies between 3.00 and 5.00, whereas the similar median gene expression values across individual samples within each phenotype evidence the integrity and consistency of the gene expression data. (**B**) The PCA plot differentiates both the phenotypes on the basis of high variable gene expression values in both phenotypes. A total of 32.1% high variable data explains a clear difference between the healthy and ARVC.

Gene expression profiling of healthy and ARVC samples resulted in genes having different expression levels across the phenotypes. Normalization of expression data resulted in high-quality data, suitable for finding significant DEGs in ARVC. The comparative distribution and variability of data between healthy and ARVC individuals are shown in Figure 1. The box plots (Figure 1A) show that each sample of both phenotypes has similar median expression values, indicating effective normalization of expression data. The principal component analysis (PCA) plot (Figure 1B) further shows a clear separation between healthy and ARVC individuals based on highly variable gene expression patterns.

**Figure 2:**
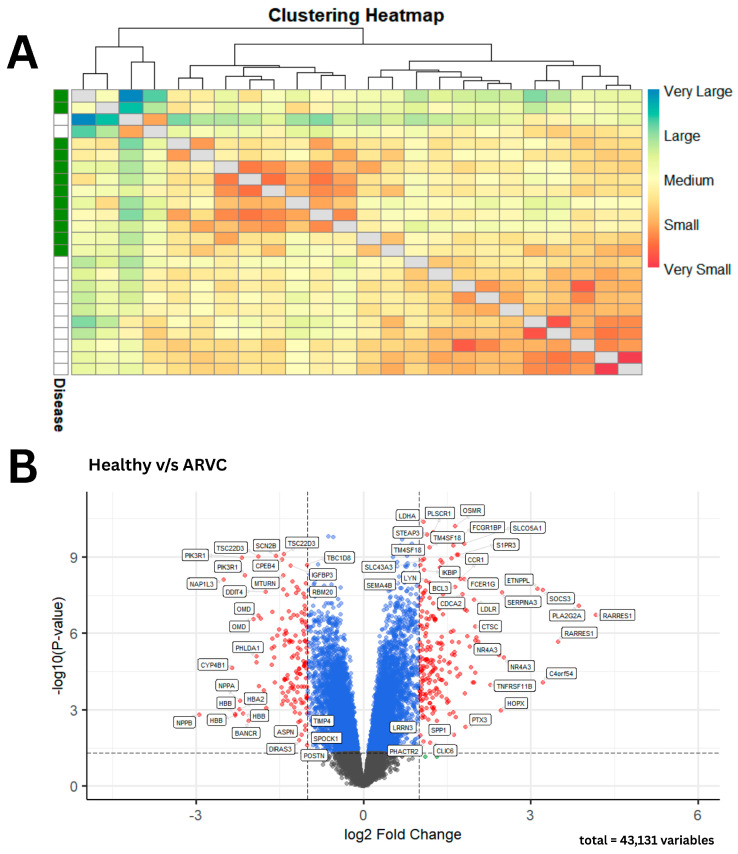
Gene expression-based clustering and differential expression in ARVC vs. healthy samples. (**A**) The heatmap reveals distinct dark-colored clusters, indicating strong similarity among samples within the same phenotype. In contrast, the lighter blue regions highlight the clear divergence in gene expression profiles between ARVC and healthy samples. (**B**) A volcano plot summarizes the significant and non-significant DEGs in ARVC with reference to healthy controls. Statistically non-significant DEGs with LogFC values below the threshold are shown as gray dots, DEGs that are statistically significant but do not meet the logFC threshold are shown as blue dots, and significant DEGs that meet the logFC threshold are presented as red dots, respectively.

A comparative analysis of phenotypes based on gene expression profiles was conducted using the Manhattan distance to evaluate intergroup similarities and differences [14]. Sample-to-sample distances were calculated for all samples within each group. The results revealed a clear distinction in gene expression profiles between healthy and ARVC samples, along with a high degree of similarity among samples belonging to the same phenotype, as illustrated in Figure 2A. DEGs analysis revealed a total of 5635 genes that showed significant differential expression in ARVC. Following the logFC threshold, 141 DEGs showed upregulation in ARVC, while 199 DEGs showed downregulation accordingly, as shown in Figure 2B.

In addition, differential expression analysis was performed separately for ARVC samples derived from the right ventricle (RV) and left ventricle (LV). As illustrated in Supplementary Tables S1 and S2, both RV-specific and LV-specific comparisons against healthy controls demonstrated highly similar differential expression patterns, with substantial overlap in the identified DEGs. Due to the absence of major transcriptomic differences between RV and LV samples, and to increase statistical power and robustness, RV and LV samples were subsequently combined and analyzed collectively as the ARVC group. The integrated analysis yielded the final set of DEGs reported in Supplementary Table S3.

### 2.2. Immune Cells Specific to Inflammation Showed Increased Proportion in ARVC Samples

**Figure 3:**
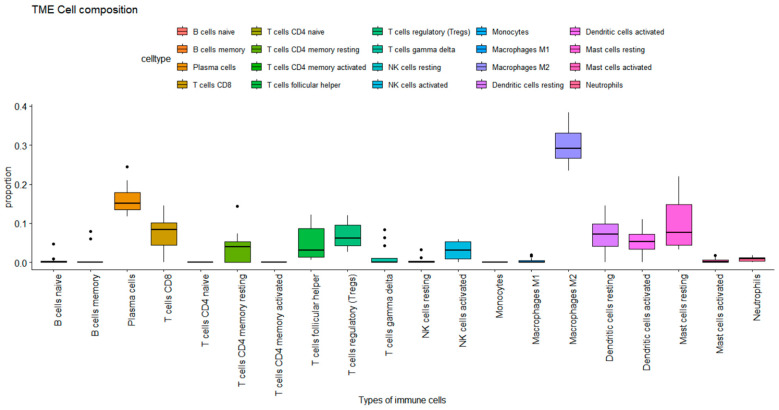
Immune cell proportions in ARVC estimated by CIBERSORT. The plot represents the proportion of immune cells in the ARVC phenotype. Each box plot represents the distribution of immune cells. The Y-axis shows the extent of proportion of immune cells, while the X-axis represents the type of immune cells. The max proportion is 1.00 while the plot reaches 0.4, showing a max of 40% proportion, where macrophages M2 showed 35% of proportion in the ARVC phenotype.

Immune cell infiltration analysis in ARVC samples showed observable immune profiles. Among the 20 immune cell types assessed, 10 were detected at significant proportions. Notably, regulatory T cells, plasma cells, CD8+ T cells, activated NK cells, resting dendritic cells, macrophages M2 cells, and resting mast cells were among the prominent infiltrating cell populations as presented in Figure 3. M2 macrophages had the highest proportion (max. 0.35), followed by plasma cells (max. 0.25), mast cells resting (max. 0.24), T cell CD8 and dendritic cells resting (max. 0.17) subsequently.

**Figure 4:**
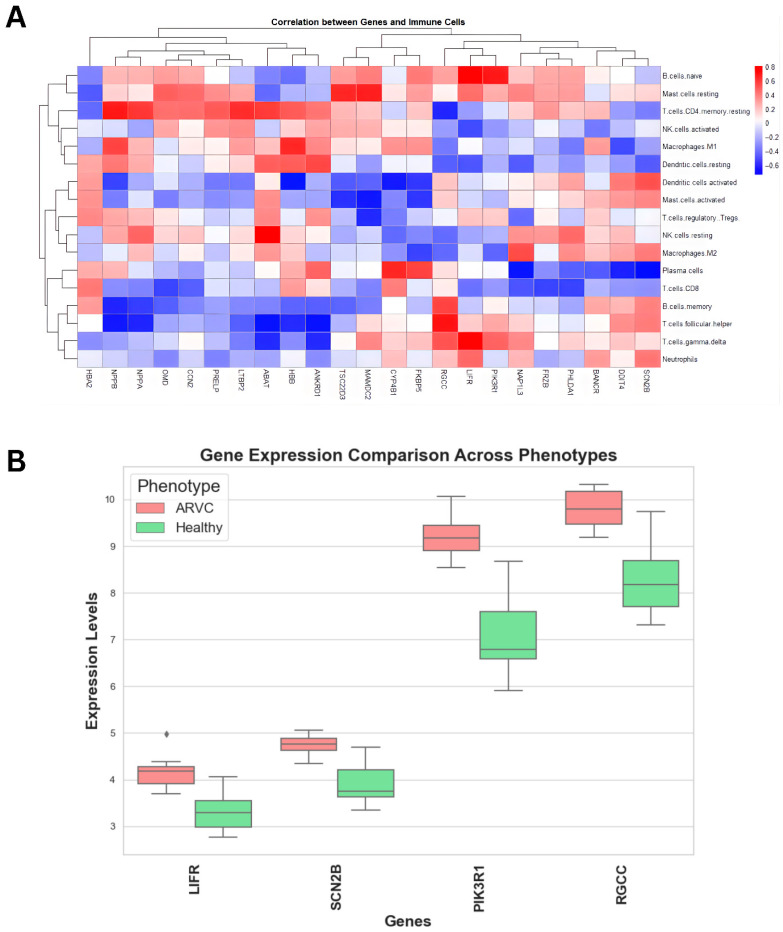
Correlation analysis of upregulated genes with immune cells. (**A**) The heatmap showed a correlation of immune cells with the upregulated genes. The key represents the type and extent of correlation coefficients. (**B**) Comparative box plots display the distribution of gene expression levels (Y-axis) for the identified genes in ARVC and healthy controls. All the genes exhibit comparatively higher expression in ARVC samples than in healthy controls.

Next, we correlate upregulated genes with the immune cells’ proportions to derive genes significantly correlated with immune cells. Upon correlating (Figure 4A), four genes showed significant correlation coefficients with immune cells. Leukemia inhibitory factor receptor (LIFR) showed a positive correlation of > 0.4 with neutrophils, T cells gamma delta, T cells follicular helper, T regulatory cells, mast cells resting, and naïve B cells. Furthermore, sodium voltage-gated channel beta subunit 2 (SCN2B) showed a correlation coefficient of >0.35 with neutrophils, T cell gamma delta, T cell follicular helper, B cell memory, macrophages M2, mast cells activated, dendritic cell activated, and mast cell resting. Similarly, regulator of cell cycle (RGCC) also showed the same correlation profile as SCN2B, while also having a positive correlation with naïve B cells. Lastly, phosphoinositide-3-kinase regulatory subunit 1 (PIK3R1) showed a correlation greater than 0.5 with T cell gamma delta and naïve B cells, and a correlation greater than 0.4 with T follicular helper cells and resting mast cells, respectively. These genes were significantly upregulated in ARVC samples compared to healthy controls (Figure 4B) and were positively associated with immune cell proportions linked to inflammation. While correlation alone does not imply causation, these statistical associations served as an initial filter to prioritize candidate genes for further functional investigation.

### 2.3. Association of PIK3R1 with Inflammation-Specific Immune Cells and Its Role in Inflammatory Pathways Makes It a Potential Candidate for ARVC Pathogenesis

**Figure 5:**
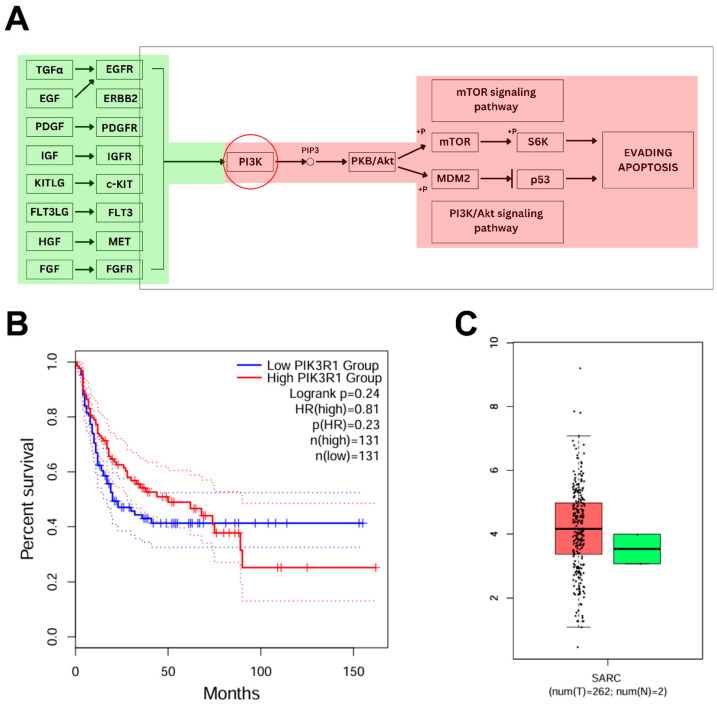
Pathway analysis of PIK3R1 and assessment of its expression and outcomes from TCGA. (**A**) KEGG pathway map illustrating the involvement of PIK3R1 in the mTOR and PI3K/AKT signaling pathways. The red-highlighted components indicate regions potentially influenced by PIK3R1 overexpression, which may dysregulate key signaling events involved in cell survival, inflammation, and apoptosis evasion, contributing to disease progression. (**B**) The plot shows the effect of increased PIK3R1 expression on patient survival, demonstrating reduced overall survival in the high-expression group. The solid lines represent Kaplan–Meier survival estimates, while the flanking dotted lines indicate the upper and lower 95% confidence intervals. The narrow spacing between the confidence interval boundaries and the main curves reflects high precision and statistical significance of the survival differences. (**C**) Box plots showed the relatively increased expression of PIK3R1 in SARC (red) as compared to healthy controls (green).

Former analysis revealed a set of genes associated with immune cells that are reported to have a significant proportion in ARVC-affected individuals. Out of it, the increased expression of PIK3R1 in ARVC samples and its positive correlation with inflammation-specific cells (T cell gamma delta, T cell follicular helper, and mast cells resting) make it observable for subsequent analysis. Kegg pathways analysis (Figure 5A) showed the direct role of PIK3R1 (PI3K) in the mTOR signaling pathway and the Akt-signaling pathway. PIK3R1, encoding the p85α regulatory subunit of class IA phosphoinositide 3-kinases (PI3Ks), is integral to the PI3K/AKT and mTOR signaling cascade. PI3K is activated downstream of several receptor tyrosine kinases, leading to the phosphorylation of phosphatidylinositol 4,5-bisphosphate (PIP2) to phosphatidylinositol (3,4,5)-trisphosphate (PIP3) and subsequent activation of AKT, which further plays significant roles in regulating cellular processes such as growth, survival, metabolism, and immune responses. In ARVC samples, PIK3R1 was significantly upregulated (logFC > 2.00), suggesting hyperactivation of the PI3K/AKT axis.

Furthermore, survival analysis based on PIK3R1 expression levels (Figure 5B) demonstrated that individuals with high PIK3R1 expression exhibited a reduced overall survival rate compared to those with low expression. In addition, reference transcriptomic data from the TCGA database revealed a markedly elevated expression of PIK3R1 in sarcoma (SARC) tissues relative to normal controls (Figure 5C), supporting a potential role of PI3K pathway dysregulation in tumorigenesis. Chronic activation of PI3K/AKT signaling promotes evasion of apoptosis (via MDM2-mediated p53 suppression), enhances angiogenesis, and facilitates uncontrolled proliferation, hallmarks of oncogenic transformation. In summary, our integrative analysis suggests that PIK3R1 plays a critical role in ARVC-associated inflammation, with potential implications for disease progression that might lead to tumorigenesis.

### 2.4. Docking of PIK3R1 with Phytochemicals

Docking studies were conducted to propose a potential therapeutic strategy aimed at mitigating the increased expression of PIK3R1, thereby reducing inflammation severity in ARVC individuals. The crystallographic structure of PIK3R1 was retrieved from the Protein Data Bank (PDB ID: 1H9O), then subjected to energy minimization, making it suitable for docking. Following minimization, all residues exhibited zero clash scores with an overall Rama score of 99.1, indicating high stereochemical quality. Domain analysis revealed that residues 3 to 107 constitute the active site of PIK3R1. Accordingly, a grid box was defined with dimensions X: 21.35, Y: 16.25, and Z: 17.31, encompassing these residues to enable targeted docking of phytochemicals into the active site of PIK3R1. Furthermore, screening of the phytochemical library yielded 98 compounds exhibiting favorable drug-likeness properties and full compatibility for potential human administration.

**Figure 6:**
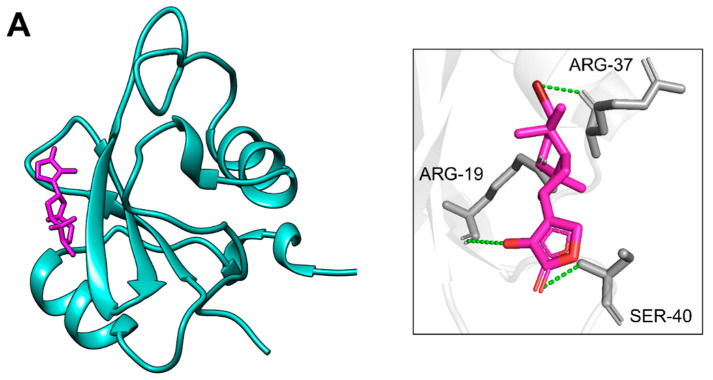

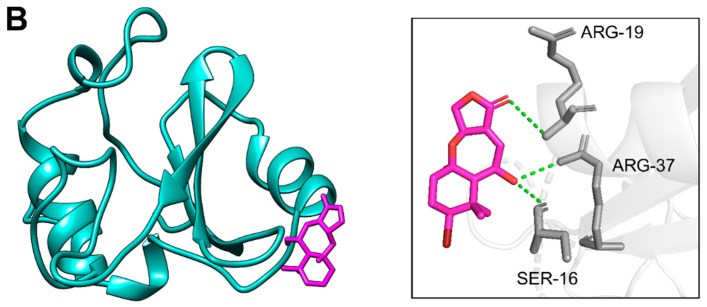
Residues of PIK3R1 creating hydrogen bonds with phytochemicals. Part (**A**) shows the residues of PIK3R1 involved in hydrogen bonds (dotted green) formation with CMNPD18967 after docking, while part (**B**) presents the interacting residues of PIK3R1 making hydrogen bonds (dotted green) with CMNPD756, respectively.

**Figure 7:**
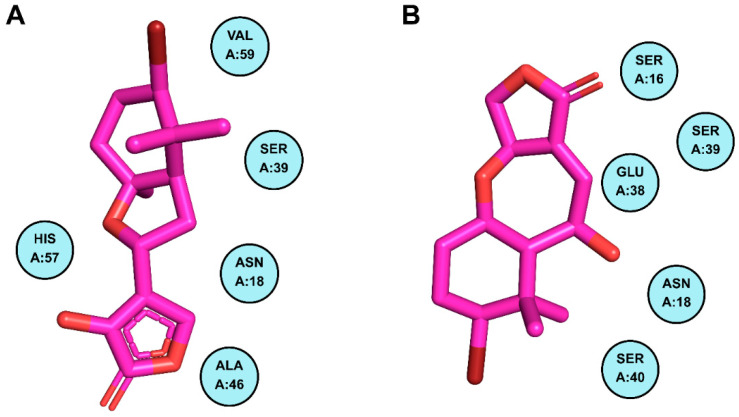
Residues of PIK3R1 creating non-bonded interactions with phytochemicals. Part (**A**) demonstrates the residues of PIK3R1 contributing to non-bonded interactions with CMNPD18967, while part (**B**) illustrates the contributing residues of the mentioned protein making non-bonded interactions with CMNPD756.

Docking of PIK3R1 with the screened compounds identified two molecules, CMNPD18967 and CMNPD756, as top candidates, exhibiting the highest binding affinities with binding energies of −5.9 kcal/mol and −5.7 kcal/mol and showcasing the drug likeness and compatible properties (Table 1). These compounds exhibited the highest binding affinities toward PIK3R1, with CMNPD18967 forming three hydrogen bonds with ARG-19, ARG-37, and SER-40 (Figure 6A), while CMNPD756 also established three hydrogen bonds with SER-16, ARG-19, and ARG-37 (Figure 6B). Non-bonded interaction analysis revealed that CMNPD18967 and CMNPD756 established multiple stabilizing contacts within the active site of PIK3R1. CMNPD18967 exhibited five key interactions involving residues ASN18, SER39, ALA46, HIS57, and VAL59 (Figure 7A), suggesting stable anchoring within the binding pocket. Similarly, CMNPD756 formed five notable interactions with residues SER16, ASN18, GLU38, SER39, and SER40 (Figure 7B), reinforcing its potential as a strong binder. These interactions, primarily involving polar and hydrophilic residues, support the compounds’ capacity to form stable complexes with PIK3R1 and highlight their therapeutic relevance.

### 2.5. Molecular Dynamic Simulations of the Docked Complex Revealed Stable Binding of Phytochemicals to PIK3R1

**Figure 8:**
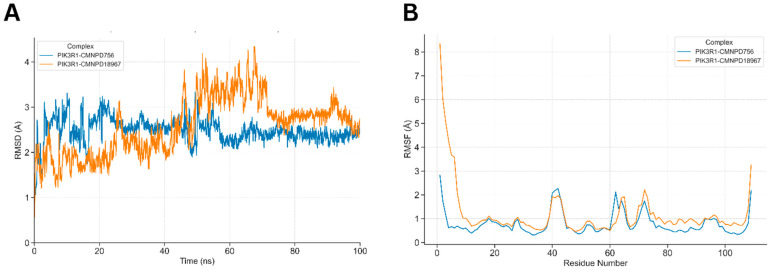
Dynamic simulations of the docked complexes. (**A**) shows the comparative RMSD of docked complexes along a 100 ns simulation run. PIK3R1-CMNPD756 RMSD increases up to 3.0 Å till 45 ns of simulation time, where after stabilizes at 2.3 Å till the end of the simulation trajectory. PIK3R1-CMNPD18967 showed notable fluctuation of RMSD till 70 ns and stabilized afterwards. (**B**) detailed comparative conformational fluctuations of each residue of both docked complexes along the simulation run, where multiple residues of both complexes showed notable conformational fluctuations at residues (see text) mainly involved in ligand binding.

Molecular dynamics (MD) simulations were conducted to assess the binding stability of the docked phytochemicals within the PIK3R1 binding pocket. In the PIK3R1-CMNPD756 complex, CMNPD756 consistently maintained its initial docked conformation throughout the simulation trajectory, indicating stable binding. In contrast, the PIK3R1-CMNPD18967 complex exhibited transient deviations at specific time points; however, CMNPD18967 regained its stability within the binding pocket over time (Figure 8A). CMNPD756 exhibited minor conformational fluctuations during the simulation, with RMSD peaks of 3.0 Å at 20 ns and 45 ns. However, it remained stable thereafter, maintaining an average RMSD of 2.3 Å until the end of the simulation. Similarly, CMNPD18967 showed a transient increase in RMSD, reaching 3.0 Å at 22 ns and peaking at 4.0 Å between 55 ns and 65 ns. Despite these deviations, the ligand eventually stabilized within the binding site, maintaining an RMSD of approximately 2.7 Å for the remainder of the simulation.

The RMSF plot illustrated the residue-wise flexibility of PIK3R1, with notable fluctuations observed in residues involved in hydrogen bonding and non-bonded interactions with the docked ligands (Figure 8B). Peaks in the RMSF profile reached up to 2.0 Å, particularly exceeding the 1.0 Å and 2.0 Å thresholds at key residues including Ser-16, Asn-18, Arg-19, Arg-37, Glu-38, Ser-39, and Ser-40. These elevated fluctuations underscore the active participation of these residues in stabilizing ligand binding through dynamic interaction patterns.

## 3. Discussion

ARVC is a rare but life-threatening cardiac disorder, with a substantial contribution to sudden cardiac deaths among young athletes and adults. The onset of ARVC is related to inherited genetic mutations, with several genes, particularly those encoding desmosomal proteins such as Plakophilin-2 (PKP2), Desmoplakin (DSP), and Desmoglein-2 (DSG2), implicated in its development [15,16]. Beyond its genetic foundation, emerging evidence underscores the pivotal role of inflammation in exacerbating myocardial damage and disease progression [17]. Several studies have identified inflammatory infiltrates in myocardial tissue from both living patients and postmortem specimens, underscoring the active role of immune responses in the ARVC phenotype [18,19]. However, the presence of inflammation in both early and advanced stages of the disease suggests that genetic predisposition alone may not fully account for the clinical variability and progression. This highlights the importance of exploring downstream molecular events, such as immune activation and inflammatory signaling, to better understand ARVC pathophysiology and uncover potential therapeutic targets.

In this context, the advancement of bioinformatics has played a transformative role in addressing such complex biological questions. It has been proven instrumental in identifying key genes and pathways involved in multifactorial diseases, enabling systematic investigation of large-scale omics data [20,21]. Numerous studies have employed bioinformatics approaches to dissect molecular mechanisms, uncover diagnostic biomarkers, and propose therapeutic targets across various inflammatory and genetic disorders [22]. Applying these tools to ARVC offers the potential to unravel its inflammatory landscape and pave the way for novel intervention strategies.

The comparative analysis of gene expression profiles between ARVC patients and healthy controls revealed significant differences between the two conditions. Furthermore, the large number of DEGs identified in ARVC samples underscores substantial alterations in transcriptional regulation associated with the disease. This widespread gene expression dysregulation likely reflects the complex pathophysiological landscape of ARVC, which extends to include active immune involvement. To further investigate the immunological component of the disease, an immune cell infiltration analysis was performed, which resulted in immune cells present in notable proportions in ARVC that provide some deeper insights into the condition. The prominence of macrophage M2 cells in ARVC might suggest a role in tissue remodeling and fibrosis within the ARVC myocardium. M2 macrophages are known to contribute to cardiac fibrosis and adverse remodeling by secreting anti-inflammatory cytokines and promoting extracellular matrix deposition [23,24]. Moreover, the notable proportion of CD8+ T cells might contribute to inflammation, as a study revealed that it can mediate cardiomyocyte injury through the release of perforin and granzyme B. Their infiltration into cardiac tissue has been associated with myocardial inflammation and damage in various cardiac diseases [25,26]. Furthermore, the presence of activated NK cells also suggests their dual role in cardiac inflammation. While they exert cytotoxic effects on infected or stressed cardiomyocytes, they also modulate immune responses by interacting with other immune cells such as dendritic cells and T cells, thereby influencing the progression and resolution of inflammatory heart disease [27,28].

Furthermore, the correlation analysis of the upregulated genes with immune cell proportions evident in ARVC also sheds light on the pathophysiology of the condition. This analysis highlights four upregulated genes that show significant correlation with the immune cells known to be associated with inflammatory responses. The positive correlation of LIFR (Leukemia Inhibitory Factor Receptor), which is known to be highly expressed in multiple malignancies, with immune cells such as neutrophils, T cells, gamma, follicular helper T cells, regulatory T cells, resting mast cells, and naïve B cells, highlights its potential immunomodulatory role in ARVC [29]. LIFR is a key component of the LIF signaling pathway and has been implicated in various oncogenic cascades, including JAK/STAT3, MAPK, AKT, and mTOR [30,31,32]. These pathways are known to influence inflammation, cell survival, and immune cell function. Its upregulation and immune associations in ARVC suggest that LIFR may contribute to disease progression by facilitating immune cell recruitment and sustaining an inflammatory cardiac microenvironment. SCN2B, which encodes the β2 subunit of voltage-gated sodium channels, primarily contributes to electrical signaling and cell adhesion. Although Wang et al. (2023) predict its association with encephalitis, its direct role in immune activation remains unclear; its correlation with neutrophils and macrophages in ARVC suggests a potential involvement in modulating cardiac immune responses [33]. Similarly, RGCC, a regulator of cell cycle progression and DNA damage response, showed associations with macrophages and B cells, implying a possible contribution to immune regulation and tissue remodeling within the ARVC microenvironment. The upregulation of PIK3R1 and its strong positive correlation with inflammation-associated immune cells also highlight its potential involvement in shaping the immune landscape of ARVC. While correlation analysis provided an initial statistical indication of gene-immune cell associations, it does not imply direct causality. To provide biological context and strengthen the inference, we integrated multiple complementary analyses, including pathway enrichment to reveal functional relevance, reference transcriptomic comparisons from the TCGA database, and survival analysis to assess clinical significance.

Pathway analysis revealed the association of the PIK3R1 gene with the PI3K/AKT/mTOR signaling pathway. This gene encodes the p85α regulatory subunit of Class IA phosphoinositide 3-kinases (PI3Ks), which are pivotal components of the PI3K/AKT/mTOR signaling pathway. This pathway is crucial for regulating a variety of cellular processes, including immune activation, metabolism, survival, and fibrosis, reflecting context-dependent functional diversity across tissues, including the heart [34,35]. Dysregulation of this pathway, especially chronic activation, has been implicated in several inflammatory cardiomyopathies, where it contributes to myocardial fibrosis, immune cell recruitment, and ventricular remodeling [36]. In the context of ARVC, elevated expression of PIK3R1 (logFC > 2.00) suggests hyperactivation of the PI3K/AKT axis, which may influence pathological cardiac remodeling through sustained inflammatory signaling. Notably, PI3K/AKT signaling also influences T cell differentiation and mast cell degranulation, both of which are key contributors to tissue damage and fibrosis in inflamed cardiac tissue [37,38]. Survival analysis further revealed that high PIK3R1 expression correlates with reduced overall survival, suggesting its prognostic relevance. Additionally, reference transcriptome analysis from the TCGA-SARC dataset showed increased PIK3R1 expression in sarcoma tissues, reinforcing its involvement in cell survival, angiogenesis, and oncogenic transformation via mechanisms such as p53 suppression and mTOR-driven proliferation [39]. Together, these findings point toward a dual role for PIK3R1; it not only acts as an upstream regulator of immune-associated inflammation in ARVC but may also drive fibrotic and potentially oncogenic processes when chronically dysregulated. These insights underscore the need for further investigation into PI3K-targeted therapies as potential modulators of ARVC progression and severity.

Phytochemicals have increasingly gained attention for their anti-inflammatory and cardioprotective effects, owing to their biocompatibility, minimal toxicity, and multi-target capacity [40]. A library of phytochemicals extracted from marine plants, screened according to their drug-likeness and Absorption, Distribution, Metabolism, Excretion, and Toxicity (ADMET) properties [41]. Furthermore, the shortlisted compounds were further evaluated using a human (hERG) inhibition prediction model [42]. hERG encodes a voltage-gated potassium channel (Kv11.1) that is essential for cardiac repolarization, and unintended blockade of this channel is a well-recognized cause of QT interval prolongation and life-threatening arrhythmias [43]. None of the investigated compounds demonstrated predicted hERG inhibitory activity, indicating a low likelihood of proarrhythmic or cardiotoxic effects. The final filtered compounds were used as inhibitory compounds against the active site of PIK3R. Out of the screened candidates, CMNPD18967 and CMNPD756 emerged as top potential binders with strong affinities, forming multiple hydrogen bonds with key residues involved in the PI3K regulatory domain.

MD simulations serve as a robust approach to validate docking predictions by examining the dynamic behavior and stability of ligand-protein interactions over time. In this study, both CMNPD756 and CMNPD18967 demonstrated persistent binding within the PIK3R1 active site over a 100 ns trajectory. CMNPD756 maintained a relatively stable conformation throughout, with minimal RMSD fluctuations, indicating strong and consistent binding affinity. CMNPD18967, although exhibiting transient deviations early and mid-simulation, ultimately stabilized, suggesting its potential to remain anchored within the binding pocket under physiological conditions. Additionally, RMSF analysis highlighted key interacting residues such as Ser-16, Arg-19, and Ser-39 with elevated flexibility, likely due to their dynamic engagement with the ligands. These findings reinforce the potential of both phytochemicals as viable PIK3R1 inhibitors, capable of maintaining stable interactions within the protein’s regulatory domain throughout dynamic physiological conditions.

## 4. Materials and Methods

**Figure 9:**
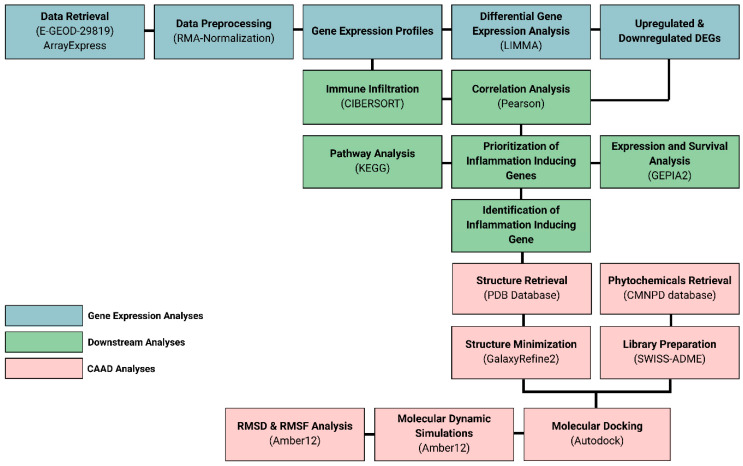
Research workflow.

The overall workflow of this study includes the identification of differentially expressed genes from a microarray dataset of ARVC phenotypes that were further processed through downstream analysis, such as immune infiltration, pathway analysis, and functional enrichment, which reveals the identification of key proteins responsible for the development and severity of ARVC in affected individuals. Further, the key player protein having the highest involvement in ARVC phenotype was inhibited through natural compounds using docking, followed by simulations, building a therapeutic hypothesis of treating the severity of ARVC in affected individuals. The overall study design is mentioned in Figure 9.

### 4.1. Retrieval of Gene Expression Data of ARVC Patients

A microarray dataset specific to the ARVC phenotype was retrieved from the Gene Expression Omnibus database using the specific keyword of “Arrhythmogenic Right Ventricular Cardiomyopathy”. A specific dataset of accession ID, E-GEOD-29819, consisted of ventricular tissue samples from six clinically confirmed ARVC patients (6 right ventricular and 6 left ventricular biopsies; total *n* = 12) and six non-failing donor hearts (healthy controls) processed similarly (6 right and 6 left ventricular samples; total *n* = 12), analyzed by Affymetrix HG-U133 Plus 2.0 arrays [44]. The gene expression profiles were obtained from cardiac tissues of both diseased and healthy individuals, where no prior therapy or medication was provided to the individuals. In the original study generating this dataset, “healthy” control samples were obtained from non-failing (NF) donor hearts that were structurally normal and intended for transplantation but not used due to technical or logistical reasons. These donor hearts showed no evidence of cardiac disease, and myocardial tissue was collected within <30 min of cold ischemia, representing the closest ethical and physiological approximation to healthy human myocardium [45].

### 4.2. Gene Expression Data Analysis

Multiple Bioconductor packages were employed in RStudio v4.4.1 for microarray preprocessing and downstream analysis. Raw expression signals were normalized using the Robust Multi-Array Average (RMA) algorithm, which standardizes intensity values across samples and minimizes technical noise [46]. Data distribution, quality assessment, and expression trends across phenotypes were visualized using the ggplot2 package v4.0.1 [47].

The statistical analysis was conducted using a Bayesian technique and a generic linear model. Using the limma v3.58.1 R package, a linear model was fitted to calculate the differentially expressed genes (DEGs) in ARVC samples in comparison to healthy samples [46]. Using the eBayes function in R, the “Empirical Bayes” model was fitted, and t-statistics were calculated, which yield the statistical significance value (*p*-value) for each DEG [48]. Significant DEGs were obtained using the adjusted *p*-value criteria of ≤0.05 and Log2 fold change (Log2FC) thresholds (Log2FC < −1 and Log2FC > 1) [49].

### 4.3. Immune Infiltration

Cell-type Identification by Estimating Relative Subsets of RNA Transcripts (CIBERSORT) analysis was used to identify immune cell proportions in ARVC samples. According to literature, the severity of ARVC has a significant positive association with inflammation. For this, identifying the proportion of immune cells in ARVC and relating it to inflammation can provide us with better insights into the role of inflammation in ARVC phenotypes. CIBERSORT used a deconvolution algorithm that has been validated on gene expression profiles measured by RNA sequencing. Further, it derives a *p*-value for the deconvolution of each sample, which provides a measure of confidence in the results where *p* < 0.05 was considered accurate for statistical significance [50].

The immune cell profiles were further specifically correlated with the upregulated genes of the ARVC samples, yielding correlation coefficients significant at the 90% confidence level (*p*-values < 0.1). Given the limited sample size and exploratory nature of this analysis, this threshold was selected to minimize the risk of Type II errors and to capture potentially meaningful biological associations. This approach enabled assessment of the relationship between ARVC-associated upregulated genes and immune cell proportions relevant to inflammatory responses.

### 4.4. Pathway Enrichment Analysis

The genes exhibiting significant positive associations with inflammation-specific immune cells were subjected to pathway enrichment analysis using the Kyoto Encyclopedia of Genes and Genomes (KEGG) database [51]. This analysis identified the involvement of these genes in specific inflammation-related pathways. Genes playing significant roles in these inflammatory pathways were selected for further exploration.

### 4.5. TCGA-Derived Data Comparison and Survival Analysis

Genes upregulated in ARVC that show significant positive associations with immune cells reported at high levels in inflammatory responses and play observable roles in pathways specific to inflammation provide significant reasons to hypothesize that these genes might play a positive role in inducing inflammation in ARVC patients. To further support our analysis, we observe the expression profiles of the selected genes in sarcoma-derived datasets present in The Cancer Genome Atlas (TCGA) database using the GEPIA2 tool, as different literature has concluded that sarcomas are mostly observed in cardiac tissues that may arise due to uncontrolled inflammatory responses [52,53]. Furthermore, we also perform disease-free survival analysis of sarcoma patients to assess the prognostic significance of these genes.

### 4.6. Structure Analysis of Target Protein

After identifying the key gene of interest associated with ARVC severity and its potential link to sarcoma, we proceeded to evaluate the inhibition of its corresponding protein through molecular docking. The three-dimensional crystal structure of the target protein was retrieved from the Protein Data Bank (PDB) and subsequently refined using the GalaxyRefine2 server to ensure structural quality prior to docking [54]. Furthermore, the InterPro database was used to predict the functional domain (active site) of the target protein responsible for its biological activity [55].

### 4.7. Library Preparation of Compounds

After obtaining a refined target protein as a receptor, we focused on phytochemical compounds to be used as ligands that could halt the functioning of the target protein. For this, a large database of phytochemicals extracted from the Comprehensive Marine Natural Products Database (CMNPD) was used [56]. First, a total of 1900+ compounds were retrieved and filtered using the SWISSADME server, which has built-in trained filtration models to exclude toxic, poorly soluble, poorly absorbed, BBB-impermeable, non-synthetic, and Pan-Assay-Interference-compounds (PAINS) compounds [41]. Furthermore, the human ether-a-go-go related gene (hERG) inhibition classification model was used to screen out compounds showing hERG inhibition [42,43]. These analyses were performed to select for the most drug-like compounds having minimal side effects in the natural body environment. The filtered compounds library was formed, which was used for docking analysis, respectively.

### 4.8. Molecular Docking

Molecular docking studies were conducted to evaluate the binding affinity of screened ligands against the target protein. Ligand structures were energy-minimized and converted to PDBQT format prior to docking. The crystallographic structure of the target protein was prepared using UCSF Chimera by removing water molecules and adding polar hydrogens to optimize the protein for docking. Grid box parameters were defined in AutoDockTools 1.5.7 based on the coordinates surrounding the functionally relevant active site residues [57]. Docking simulations were executed using PyRx v0.8 with Autodock Vina, and protein–ligand interactions and docking conformations were further analyzed and visualized using Discovery Studio v21.1 [58].

### 4.9. Molecular Dynamic Simulations

Molecular dynamics (MD) simulations were performed for the docked complexes using Amber v22 [59]. The target protein was modeled using the AMBER FF19SB force field, and the RESP fitting method was applied to acquire the charges of the inhibitor [60,61]. The global AMBER force field 2(GAFF2) was used to create topology preparation files for ligands using the Antechamber module in AmberTools12 [62]. The complexes were subsequently solvated in a truncated octahedral box of TIP3P water molecules with a buffer size of 12 Å after being neutralized with 10 Na+ ions [63]. Amber12 was utilized to assign default protonation states to protein residues. Energy minimization and molecular dynamics (MD) simulations for each system were conducted using the SANDER module of the Amber12 package. Initially, 10,000 minimization cycles were performed on all atoms in the system, comprising 700 steps of steepest descent followed by 9300 steps of conjugate gradient, to eliminate unfavorable steric interactions and approach an energy minimum. After energy minimization, position restraints were applied at constant volume (NVT) for 100 ps with a force constant of 10 Kcal/mol at 100 K temperature, followed by constant pressure (NPT) for 100 ps with a force constant of 1 Kcal/mol at 300 K temperature. Ultimately, a production run lasting 100 ns was executed at constant pressure (NPT ensemble) using a 2 fs time step and isotropic position scaling (ntp = 1) at 300 K. Parameters such as Root Mean Square Deviation (RMSD) and Root Mean Square Fluctuation (RMSF) were calculated to assess the binding stability and flexibility patterns of the docked complexes.

## 5. Conclusions

Although ARVC is a genetically driven cardiomyopathy, increasing evidence suggests that inflammatory signaling contributes to disease progression and ventricular remodeling. By integrating differential expression profiling, immune infiltration analysis, and pathway enrichment, this study identifies PIK3R1 as a potential molecular driver of inflammation in ARVC. Its significant upregulation, strong association with inflammation-linked immune cell populations, and involvement in PI3K/AKT/mTOR signaling indicate that PIK3R1 may influence immune activation, tissue damage, and fibrofatty replacement characteristic of ARVC.

Beyond defining a putative pathogenic mechanism, these findings narrow the biological search space and provide a focused candidate for future diagnostic, prognostic, and mechanistic studies. Computational docking and molecular dynamics simulations further highlight two marine-derived phytochemicals, CMNPD756 and CMNPD18967, as preliminary inhibitory candidates capable of stably interacting with PIK3R1, offering a starting point for therapeutic exploration. However, the anti-inflammatory effects of PI3K/AKT pathway inhibition require further experimental and clinical validation to confirm therapeutic relevance. In addition, targeting PI3K/AKT signaling must be approached cautiously, as its inhibition can potentially disrupt essential proliferative and metabolic processes, particularly in insulin-responsive tissues.

Overall, while these findings provide a strong hypothesis-generating framework, experimental verification in cellular, animal, and patient-level systems remains essential before translational application can be considered.

## Figures and Tables

**Table 1:** Properties of shortlisted docked phytochemicals. The table demonstrates the physical and biological properties of top-docked phytochemicals. # means total number of violations.

CMNPD ID	CMNPD756	CMNPD18967	Structure
Formula	C15H21BrO4	C15H21BrO4	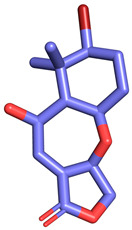
Molecular Weight	345.23	345.23	
Hydrogen Bond Donor	1	1	
Hydrogen Bond Acceptor	4	4	
Heavy atoms	20	20	
Lipophilicity	2.25	2.65	
BBB-permeability	Yes	Yes	
GI-absorption	High	High	
Pgp substrate	No	No	
CYP1A2 inhibitor	No	No	
CYP2C19 inhibitor	No	No	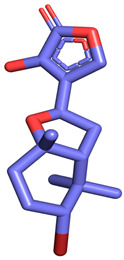
CYP2C9 inhibitor	No	No	
CYP2D6 inhibitor	No	No	
CYP3A4 inhibitor	No	No	
hERG Blocker	No	No	
Lipinski #violations	No violations	No violations	
Ghose #violations	No violations	No violations	
Veber #violations	No violations	No violations	
Bioavailability Score	0.55	0.55	
PAINS #alerts	0 alerts	0 alerts	
Lead likeness #violations	1	1	
Synthetic Accessibility	5.02	4.59	

## Data Availability

The data generated in the work is presented in the manuscript. All the raw data generated in the manuscript is provided as Supplementary and uploaded along with the manuscript.

## Supplementary Materials

The following supporting information can be downloaded at https://www.mdpi.com/article/10.3390/ph18121873/s1: Table S1: DEGs across ARVC (Right Ventricle) with reference to Healthy Controls. Table S2: DEGs across ARVC (Left Ventricle) with reference to Healthy Controls. Table S3: DEGs across ARVC (Right Ventricle + Left Ventricle) with reference to Healthy Controls.
